# Mitochondrial Redox Signaling and Tumor Progression

**DOI:** 10.3390/cancers8040040

**Published:** 2016-03-25

**Authors:** Yuxin Chen, Haiqing Zhang, Huanjiao Jenny Zhou, Weidong Ji, Wang Min

**Affiliations:** 1 The First Affiliated Hospital, Center for Translational Medicine, Sun Yat-sen University, Guangzhou 510080, China; yuxin_chen2015@163.com (Y.C.); qingzh123@163.com (H.Z.); wdji2008@126.com (W.J.); 2 Interdepartmental Program in Vascular Biology and Therapeutics, Department of Pathology, Yale University School of Medicine, 10 Amistad St, New Haven, CT 06520, USA; huanjiao.zhou@yale.edu

**Keywords:** mitochondria, redox signaling, cancer progression, apoptosis, anti-oxidant proteins

## Abstract

Cancer cell can reprogram their energy production by switching mitochondrial oxidative phosphorylation to glycolysis. However, mitochondria play multiple roles in cancer cells, including redox regulation, reactive oxygen species (ROS) generation, and apoptotic signaling. Moreover, these mitochondrial roles are integrated via multiple interconnected metabolic and redox sensitive pathways. Interestingly, mitochondrial redox proteins biphasically regulate tumor progression depending on cellular ROS levels. Low level of ROS functions as signaling messengers promoting cancer cell proliferation and cancer invasion. However, anti-cancer drug-initiated stress signaling could induce excessive ROS, which is detrimental to cancer cells. Mitochondrial redox proteins could scavenger basal ROS and function as “tumor suppressors” or prevent excessive ROS to act as “tumor promoter”. Paradoxically, excessive ROS often also induce DNA mutations and/or promotes tumor metastasis at various stages of cancer progression. Targeting redox-sensitive pathways and transcriptional factors in the appropriate context offers great promise for cancer prevention and therapy. However, the therapeutics should be cancer-type and stage-dependent.

## 1. Introduction

Mitochondria are the organelles to produce energy via oxidative phosphorylation (OXPHOS), which play essential roles in multiple fundamental cellular events, including fatty acid synthesis, calcium homeostasis, and apoptotic signaling [1,2]. Interestingly, cancer cells shift their energy production away from OXPHOS toward glycolysis during malignant progression, even when aerobic metabolism is available (a phenomenon known as the Warburg effect). Warburg proposed the damaged mitochondria theory over 80 years ago [3], and this led to the hypothesis that mitochondrial dysfunction could be the cause of cancer. However, it was later realized that mitochondrial respiration is operational in cancer cells [4]. Although cancer cells switch to fermentation instead of oxidative respiration, it may not be the pivotal cause of cancer. It is now accepted that mutations in oncogenes and tumor suppressor genes are responsible for the malignant transformations [5]. Thereafter, cancer cell can reprogram their energy production, by limiting their energy metabolism largely to glycolysis. This switch from oxidative respiration to glycolysis is beneficial to cancer cells in that it accommodates energy and substrates required for the fast proliferation. Moreover, cancer-associated mutations have been identified in gene encoding for several metabolic enzymes such as succinate dehydrogenase (SDH), fumarate hydratase (FH), and isocitrate dehydrogenase (IDH), all enzymes of the tricarboxylic acid cycle (TCA cycle) in mitochondria [6,7]. Recent studies suggest that that cancer cells rewire mitochondrial respiration, redox signaling and metabolic fluxes to support enhanced anabolic reaction. Specifically cancer cells reprogram glycolytic fluxes and mitochondrial metabolism to decouple ATP production from electron transfer chain (ETC) complexes, but couple the Krebs cycle with ETC complexes hence ROS signaling. Several excellent reviews have been focused on the role of metabolic reprograming in cancer development [8,9,10,11,12].

During mitochondrial respiration, redox reactions occur, the production of mitochondrial ROS are highly regulated by multiple metabolic and redox sensitive pathways. Recent study indicated that reverse electron transport from complex II to complex I is likely to be a major pathway for mitochondrial ROS production, and this activity could be regulated by redox-sensitive mitoK_ATP_ and mitochondrial ATP level [13]. On one hand, low level of ROS functions as signaling messengers promoting transcriptional activation and normal cell proliferation, such as nuclear factor-κB (NF-κB) and activation protein-1 (AP-1). On the other hand, excessive ROS induced oxidative stress is detrimental to the normal function of cellular lipids, proteins, and DNA. Excessive ROS could induce DNA mutation, facilitating tumor initiation. ROS also promotes tumor cell metastasis, depending on tumor types and stages. In some cases, excessive ROS cause tumor cell death through apoptosis or necroptosis pathways [14]. While many apoptotic proteins are present in mitochondrial intermembrane, such as cytochrome c [15] and apoptosis-inducing factor (AIF) [16], increased mitochondrial ROS induces apoptosis via releasing proapoptotic factors followed by caspase activation. Mitochondria also play a role in necroptosis. Specifically, ROS-activated RIP3/RIP1 kinases phosphorylates mixed lineage kinase domain-like protein (MLKL) and mitochondrial phosphoglyceratemutase long form (PGAM5L) and engage a short form PGAM5S on the mitochondrial membrane to induce mitochondrial fission and necroptosis [17]. Oxidative stress has been linked with the development and progress of various diseases such as autoimmune disorders, cardiovascular, neurodegenerative diseases and cancer. In the context of carcinogenesis, it is established that ROS is one of the most important and well-characterized factors that leads carcinogenesis, tumor development and progression by redox signaling pathways [18,19]. Indeed, ROS has been shown to closely link with each stage of tumor initiation, development and progression [20,21]. During the initiation stage, ROS may produce DNA damage by introducing gene mutations and DNA structural alterations, further promoting oncogenic transformation [22]. In the promotion stage, whether ROS promotes tumor cell survival or death depends on the context of cells and tissues, the location of ROS production, and the concentration of each ROS [23]. During the tumor proliferation stage, the induction of redox-sensitive pathways is essential as large amount of energy is required for cell division, whereas excessive metabolic by-product from mitochondrial respiratory chain needs to be eliminated to prevent oxidative stress and subsequent cell death. Finally, redox status also regulates cell migration and further facilitates tumor invasion and metastasis process, majorly through cell adhesion protein [24] and chemokines [25,26]. Furthermore, ROS is an important stimulus of angiogenic signaling and stimulates the growth of new blood vessels, which is critical for tumor growth and metastasis [20,27,28,29,30]. Besides, a critical connection between redox and metabolic pathway in cancer cells is hypoxia-inducible factor 1α (HIF-1α) pathway. Mitochondrial respiration under prolonged hypoxic conditions results in increased ROS, which is essential for upregulation of HIF-1α pathway. HIF-1α mediates a metabolic switch from oxidative phosphorylation to lactate fermentation and controls glycolytic gene expression, further supporting cancer cell survival [31]. This phenotype seems to be recapitulated by loss of Sirtuin-3 (Sirt3), a mitochondrial form of lysine deacetylase in tumor cells. It has been shown that mouse tumors lacking sirt3 exhibit high levels of ROS that induces genomic instability, and increase HIF-1 protein levels, which in turn reprograms cellular metabolism and increases glucose consumption (Warburg effect) [3]. Therefore, Sirt3 acts as a fidelity protein and a tumor suppressor gene within the mitochondria. Interestingly, it is found that low expressed antioxidants and dysregulated ROS downregulates Sirt3 gene expressions [32]. It is convincible that ROS may downregulate sirt3 expression in cancer cells, promoting cancer progression. Indeed, Sirt3 expression is decreased in many different human cancers. Taken together, Sirt3 mechanistically connects mitochondrial ROS, metabolism reprogramming and cancer progression.

To preserve the well-coordinated and balanced redox homeostasis within the mitochondria, the human body lines up a system to defend against oxidative stress by both endogenous antioxidant system and exogenous antioxidants supplied by food. Many redox couples function in mitochondrial to detoxify ROS and regulate redox-sensitive process, including manganese superoxide dismutase (MnSOD or SOD2) [33], mitochondrial glutaredoxin (Grx2) [34], glutathione peroxidase (Gpx) [35], and thioredoxin 2 (Trx2) system [36]. Here we review the above key mitochondrial antioxidant systems, discuss their redox signaling pathways, and the role they play in tumor development and progression. Most of them are indispensible for fundamental cellular functions, embryonic development and appropriate cardiovascular function. Further, their expressions are modulated in many cancer cell types, and they can further trigger downstream critical transcriptional factors and mediate post-translational modifications. Of note, regulation of these interconnected mitochondrial redox pathways is as a result of mitochondrial reprograming [37]. Deep understanding of each antioxidant system mediated downstream signaling pathway in the context of different cancers help develop comprehensive effective anti-cancer therapeutic strategies.

## 2. Manganese Superoxide Dismutase (SOD2)

Manganese superoxide dismutase (MnSOD or SOD2) is an antioxidant enzyme localized in mammalian mitochondrial matrix [38]. It is encoded by the nuclear SOD2 gene and further transported to the mitochondria via a targeting sequence. SOD2 helps eliminate free radicals in the mitochondria by catalyzing the conversion of the superoxide anion (O_2_^−^) to hydrogen peroxide (H_2_O_2_) [39]. Around 0.1%–0.3% of the O_2_ reduced through the mitochondrial respiratory chain is transformed in O_2_^−^, which is dismutated by SOD2. In addition to enzymatic function of eliminating superoxide, SOD2 might also function as a signaling regulator in stress-induced adaptive protection via anti-apoptotic pathways. SOD2 is found to impact the activity of transcriptional factors (such as NF-κB, hypoxia-inducible factor 1 (HIF-1), AP-1, and p53) [40] that regulate cell transformation, proliferation, and angiogenesis. SOD2 plays a fundamental role in the regulation of cell proliferation [41,42,43]. Not surprisingly, SOD2 expression is essential for survival, as deletion of the SOD2 gene in mice resulted in death within five days after birth [33], along with severe dilated cardiomyopathy and accumulated lipid in the liver and muscles. A similar study also observed that SOD deficient mice that only survive up to three weeks of age show multiple pathologic phenotypes, including degenerating neurons and cardiomyocytes with impaired mitochondrial injury [44].

**Figure 1:**
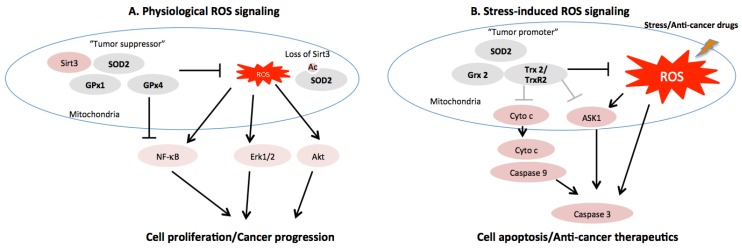
The redox signaling pathways of several key mitochondrial antioxidant systems and their roles in cellular apoptosis and proliferation. (**A**) Basal mitochondrial ROS activate cell survival and proliferation signaling to promote cancer progression. Mitochondrial anti-oxidant proteins could scavenger basal ROS and function as “tumor suppressors”. Deacetylase Sirt3 deacetylates SOD2 and enhances its activity. Loss of Sirt3 promotes oncogenesis, in part, by diminishing SOD2 activity; (**B**) Tumor micro-environmental stresses such as cytokines and anti-cancer drugs stimulate excessive ROS to activate apoptotic signaling. Under these settings, the mitochondrial anti-oxidant proteins could limit excessive ROS-induced apoptosis to act as “tumor promoter”.

The role of SOD2 in the context of cancer is still controversial. It is suggested that the SOD2 biphasically modulates the cancer cells depending on its concentration and the specific context [23] (Figure 1). In human breast cancer cell (MCF-7) and rat glioma cells, overexpression of SOD2 also inhibits the malignant phenotype of several human tumor cell lines [45,46], possibly through inhibition of AP-1 and NF-κB [47,48]. However, a lower SOD2 activity is detected in tumor cells from many reports, while some studies also reports an elevated SOD2 level in tumor cell lines [49]. The cancer cell might have reduced SOD2 activity at the tumor early stage but switch to higher SOD2 activity later during cancer progression [50,51]. Overexpression of SOD2 protects mouse epithelial tissues from apoptosis by maintaining the mitochondrial membrane [52], enhances TNF signaling [53], majorly via removal of excessive oxidative stress [54,55]. The exact mechanism underlying SOD2-mediated cell growth alteration, especially under stress-induced adaptive response, remains to be elucidated. One recent exciting study suggested that clearance of mitochondria superoxide is capable of selectively inhibiting redox-sensitive survival and metabolic pathways. Using mitochondria superoxide scavenger, (2-(2,2,6,6-tetramethylpiperidin-1-oxyl-4-ylamino)-2-oxoethyl) triphenylphosphonium chloride (mitoTEMPO), diminished mitochondrial superoxide could inhibit cell growth, viability and induce apoptosis in melanoma cells, but not nonmalignant skin fibroblasts. This provides a novel mitochondrial-targeted therapy strategy with reduced cytotoxic side effect [56].

Another key feature of SOD is the presence of lysine 122 that could be deacetylated by a primary mitochondria deacetylase, Sirtuin 3 (Sirt3) [57,58]. Once lysine 122 is deacetylated, the enzymatic activity of SOD is increased, followed by decreased cellular ROS, and prevention of stress-induced genomic instability. Further, Sirt3 also controls glycolytic metabolism by regulating stability and activity of HIF1α [59]. By this way, Sirt3 regulates activities of mitochondrial complex and modulates fatty acid metabolism, an important cross-talk between ROS and mitochondrial metabolic pathways. Indeed, loss of Sirt3 exhibited continuous steady state levels of ROS and oxidative stress [60,61,62]. Sirt3^−/−^ mouse embryonic fibroblasts (MEFs) showed a stress-induced genomic instability and were highly sensitive with oncogenes [60]. Overexpression of Sirt3 *in vivo* was capable of attenuate tumorigenesis in xenografts, even after tumor initiation [63]. Sirt3 deficient mice developed estrogen- and progesterone-(ER/PR−)-positive mammary tumors [60]. Consistently, cohort study showed heterozygous loss of SIRT3 in 40% of human breast cancers (11) formed in O_2_ [60] and decreased Sirt3 expression in different types of human cancers. All the above evidence suggests that a deacetylase that activates mitochondrial antioxidant can function as a tumor suppressor.

## 3. Mitochondrial Glutaredoxin-2

Glutaredoxin-2 (Grx2) is a thiol-disulfide oxidoreductase that catalyzes the reversible interactions of protein thiols with the mitochondrial glutathione pool [64]. It is a key player in redox signaling via reversible posttranslational modification of protein thiols. Based on sequences of active site motif, Grx2 can be grouped by dithiol Grxs with a Cys-X-X-Cys active site and monothiol Grxs with a Cys-X-X-Ser active motif. Dithiol Grxs are capable to reduce disulfides by either using both cysteines within the active site motif or solely via active site cysteine at N terminus [65,66]. Currently, the catalytic function of monothiol Grxs remains elusive. The thiol oxidoreductase activity of Grx2 has been shown to be indispensable for proper mitochondrial function in vascular formation and cardiovascular disease. S-glutathionylation of complex I by glutathione correlates with the loss of complex I activity and Grx2 could reverse this effect by deglutathionylation of complex I in mitochondrial membranes [67]. Increases and decreases in 2GSH/GSSG prompt Grx2-mediated deglutathionylation and glutathionylation of complex I, respectively, which then changes its activity and superoxide production [68].

Grx2 has been shown to exert a protective role against apoptotic and oxidant stress. Overexpression of Grx2 in mouse heart protects from doxorubicin and ischemia-induced cardiac injury [69] and reduces infarct formation in heart tissue [30]. Conversely, Grx2 knockout induces left ventricular hypertrophy and localized fibrosis in mice [70]. In a zebrafish model and various types of cultured cells, it is shown that Grx2 plays a pivotal role in brain development, and the depletion of Grx2 leads to loss of neurons through cell apoptosis. Furthermore, Grx2 also affects the embryonic angiogenesis, as deglutathionylation of a single cysteine in the protein sirtuin 1 modified by Grx2 is required for formation of a functional vascular system [71]. This suggests that the thiol redox signaling could also facilitate angiogenesis in tumor. In tumor cells, cells with decreased Grx2 are dramatically sensitized to cell death induced by anti-cancer drugs [72]. Overexpression of Grx2 inhibits cytochrome c release and caspase activation induced by intracellular ROS [73]. Collectively, these studies highlight the important anti-apoptotic function of thiol redox signaling mediated by Grx2 in the context of various disease models including cancer (Figure 1).

## 4. Glutathione Peroxidase 1 (GPx-1) and Phospholipid Hydroperoxide GPx (GPx-4)

Glutathione peroxidase (GPx) is a selenocysteine-dependent glutathione peroxidase capable of metabolize hydrogen peroxides and phospholipid hydroperoxides. Hydrogen peroxides and lipid hydroperoxides have been shown to activate growth factor receptor (e.g., EGFR) directly [74], result in Akt and ERK1/2 phosphorylation [75]. There are eight GPx isoenzymes identified in mammals so far. Among them, only GPx1 and GPx4 are found in mitochondria, but they are also found in other organelles within cells.

GPx1 is the major enzyme responsible for removing soluble peroxide ubiquitously found in the cytosol and mitochondria of all cell types. GPx1 acts as a tumor suppressor gene via several potential mechanisms. By regulating peroxide tone and reducing ROS, GPx1 overexpression diminishes mitochondrial-dependent protein disulfide bond formation and reduces mitochondrial potential, further decreases ATP generation and attenuates oxidative-mediated growth factor receptor transactivation [76] and insulin receptor activation of Akt [77], leading to reduced cellular proliferation. GPx1 overexpression also suppresses the intracellular ROS [78] and therefore exhibits a critical anti-cancer role of GPx1 in carcinogenesis (Figure 1). Recent study suggests that glutamate dehydrogenase 1 (GDH1), a mitochondrial enzyme critical for redox homeostasis, is shown to control the intracellular levels of α-ketoglutarate and further metabolite fumarate, which binds to and activate GPx1 in cancer cells. Activation of GPx1 results in imbalanced redox homeostasis and, more importantly, attenuates cancer cell proliferation and tumor growth [79]. Single-nucleotide polymorphism (SNP) meta-analysis in a case controlled study shows GPx1 Pro198Leu polymorphism is associated with a significantly increased risk of bladder cancer compared to GPx1 Pro/Pro genotype [80]. *In vitro*, animal studies, and human genetics all suggests that decreased levels of GPx-1 is correlated with increased risk of cancer.

Different from other GPxs, GPx4 is the only glutathione peroxidase that preferentially accepts phospholipid hydroperxoides in membrane as oxidizing substrate. GPx4 is synthesized as a long form (23 kDa) and a short form (20 kDa). The long form of GPx4 localizes in mitochondrial as it is a mitochondrial signal peptide, while the short GPx4 is found in other organelle of cells. With normal cells, GPx4 is essential to prevent RIP3 dependent necroptosis in erythroid precursor cells [81]. Furthermore, GPx4 inactivation in mice and cells shows 12/15-lipoxygenase-derived lipid peroxidation, triggering AIF mediated cell death [82]. Systemic deletion of GPx4 in mice causes embryonic lethality, which is not observed when other GPx genes are deleted [83], indicating a unique role for GPx4 in embryonic development. Conditional GPx4 knockout mice die with lipid peroxide generation [82,84], highlighting the significance of GPx4 for protecting cells from detrimental effects of lipid peroxides. In the context of cancer, however, GPx4 also appears to be a tumor suppressor in the majority of cases (Figure 1). GPx4 expression is decreased in pancreatic and breast cancer cells [85,86]. Overexpression of Gpx4 leads to reduced tumor growth of weakly tumorigenic L929 fibrosarcoma cells and pancreatic cancer cells [87], possibly mediated by inhibition of NF-κB activation [88]. However, recent evidence suggests that GPx4 deletion also led to loss of diffuse large B cell lymphomas and renal tumor cell possibly mediated by ferroptosis [89]. A genetic variant in gene encoding GPx4 has been reported to influence colorectal cancer risk [90]. However, the exact mechanism of interplay of Gpx4 and specific tumor host microenvironment is still under investigation.

## 5. Thioredoxin 2 System

Trx2 belongs to thioredoxin (Trx) superfamily of thiol-disulfide oxidoreductases. Mitochondria-specific Trx2 system consists of Trx2, Trx2 reductase (TrxR2), and Trx2-dependent peroxidase (Prx3). Thioredoxin 2 (Trx2) is first identified in mitochondrial of rat liver cell as early as 1996. Human homologue Trx2 is a small redox protein with 166 amino acids along with a conserved active site of Trp-Cys-Gly-Pro-Cys-Lys [91]. Trx2 has two redox-active sites (C90 and C93). Trx2 is able to undergo reversible oxidation to Cys disulfide (Trx-S2), via the transfer of reducing equivalents from the catalytic site Cys residues to a disulfide protein substrate (protein-S2), thus maintains protein in a reduced state. TrxR2 was considered as the only known enzyme catalyzing Trx2 reduction in mitochondria [36,92], but recent evidence shows that Grx2 could also protect Trx2 from oxidation [93]. Prx3 is a thioredoxin peroxidase with a mitochondrial signal sequence, which utilizes Trx as specific and super-fast electron donor for the reduction of H_2_O_2_ generated by mitochondria metabolism [94]. Trx2 system is ubiquitously expressed, especially highly observed in metabolically active tissues of the heart, brain and liver [95].

Trx2 regulates the activity of many well-characterized transcription factors and apoptosis signaling factors, such as NF-κB [96] and apoptosis signal-regulating kinase 1 (ASK-1) [93] through modulating the redox-regulatory disulfides of the protein. As Trx2 can reduce disulfide bonds, overexpression of Trx2 is able to increase mitochondrial membrane potential and ATP synthesis [97]. The deficiency of Trx2 leads to cytochrome c release from mitochondria and activation of caspase 3 and 9 (Figure 1). This could be due to the fact that Trx2 help maintain the Bcl-xL protein level and regulate the mitochondrial outermembrane permeabilization and apoptosis by redox-active cysteine-independent mechanism [98]. In Hela cells, Trx2 is able to detoxify TNF-induced increased mitochondrial ROS and apoptosis, while C93S dominant negative Trx2 fails to do so [93,96]. This is the first evidence indicating that beyond GSH, there is an alternative antioxidant system that also regulates TNF-mediated redox signaling in mitochondria. Besides, Trx2 also attenuates activities of PI3K/Akt, p70S6K and eIF4e and can inhibit hypoxia-evoked HIF-1α, a redox-sensitive transcription factor [99]. In addition to transcriptional modulation, Trx2 is also able to participate in post-translational modifications. For example, Trx2 mediates Fas-induced denitrosylation of mitochondrial caspase-3, a process required for caspase-3 activation, which further facilitates apoptosis [100].

Given multiple cellular functions observed from Trx2, it is not surprising to find that global knockout of a single gene of anti-oxidant system (Trx2 or TrxR2) leads embryonic lethality. The homozygous Trx2^−/−^ embryos die after implantation at Theiler stage 15/16. Massive apoptosis of open anterior neural tube are observed at 10.5 days postcoitus, and the timing coincides with the maturation of mitochondria, which could be due to increased oxidative damage [101]. Trx2^+/−^ mice are fertile but with elevated oxidative stress to nuclear DNA, lipid, and protein in the liver. Further, increased apoptosis in liver is also observed from Trx2^+/−^ mice [102]. This suggests that Trx2 not only protects against oxidative stress in mitochondria, but also makes the cells more resistant to ROS-induced apoptosis *in vivo*.

Endothelium cell is one of the vascular cells that lines blood vessels, and its proliferation and migration is vital for angiogenesis as well as tumor survival and metastasis. In endothelial cells, we identify that mitochondrial ASK1 is able to bind to Trx2 [93,103]. Cys-30 in the N-terminus ASK1 is critical for binding of Trx2. Mutation of ASK1 at Cys-30 sensitizes ASK1-induced apoptosis. Consistently, overexpression of Trx2 inhibits ASK1-induced apoptosis. Furthermore, upon mitochondrial ASK1 releases from Trx2, ASK1 mediates a novel apoptotic pathway distinct from that induced by TNF. Because the above events do not have any impact on JNK activation, Bid cleavage, and Bax translocation, a classical apoptotic signaling cascade induced by TNF. Further, in femoral artery ligation model, we also identify that Trx2-overexpressing EC exhibits increased resistance to oxidative stress-induced apoptosis. This is mediated by two parallel pathways—detoxifying ROS to increase NO bioavailability and inhibiting ASK1 activity [104]. Besides endothelium cells, Trx2 also prevents the apoptosis of cardiomyocytes. Ablation of cardiac-specific Trx2 exhibits the disorganized mitochondrial arrays and swelling, impairs ATP production and reduces respiration. Further, global gene perturbation is observed with deregulated apoptotic-related genes, such as two-fold increase for pro-apoptotic Fas-Ligand and declined level of anti-apoptotic Bcl2 molecule, revealed by NanoString analysis [105]. Again, ASK1 is identified as a downstream effector to leading to cardiomyocyte apoptosis. This demonstrates that ASK1 is essential for Trx2 deletion induced mitochondrial dysfunction, excessive ROS production, and apoptosis in cardiomyocytes *in vitro*. Indeed, the myocardium of patients with dilated cardiomyopathy (DCM) shows reduced Trx2 expression compared to that of organ donors with preserved cardiac function. Further, phosphorylated ASK1 and active caspase-3 are increased in idiopathic DCM patients, indicating human Trx2 is also critical to inhibit ASK1-dependent apoptosis signaling.

Increased evidence suggests that Trx system might be a new target for anticancer drug development. First, TrxR, Trx, and Prx are overexpressed in many aggressive tumor [106], and the tumor cells appear more dependent on a Trx system perhaps for the constant requirement of DNA synthesis. Indeed, TrxR2 is reported to be a potentially clinically relevant target, inhibition of which is critical for proteasome inhibitor-dependent cytotoxicity, oxidative stress and Endoplasmic reticulum (ER) stress [107]. In multiple myeloma, the proteasome inhibitors bortezomib (BTZ) and carfilzomib (CFZ) transcriptionally repress TrxR2, and lead to increased oxidative stress and ER stress. The reconstitution of Trx2 in BTZ or CFZ treated multiple myeloma cells are able to reduce oxidative stress, ER stress and cell death. Thereby, the inhibition of TrxR2, and possible Trx2, might be a promising strategy for MM therapy [107]. Another study shows that Organogold (III) complexes, emerging as a novel class of metal complexes as an effective inhibitor of TrxR2, improves anti-proliferative effect and higher apoptosis rates compared to the other anti-tumor drugs [108]. Similar to TrxR2, the overexpression of Prx3, another member from mitochondrial-specific Trx2 family, is also able to protect cancer cells against apoptosis induced by hypoxia and anticancer drug such as imexon [109]. However, the role of Trx2 in tumor development is not well studied at this moment. One preliminary study finds that Trx2-transgenic mice have a slightly higher incidence of cancer than wild-type mice at old age (24–26 months) [110]. This is consistent with *in vitro* finding that increased Trx2 expression attenuated apoptotic signaling pathways.

The Trx2-TrxR2 system has also been shown to be an anti-angiogenic target. Auranofin, a gold compound initially developed for treating rheumatoid arthritis, recently is shown also beneficial for cancer therapy. Vascular endothelial growth factor receptor-3 (VEGFR3) is an EC surface receptor essential for angiogiogenesis and lymphangiogenesis, which is further identified as a novel target of auranofin by our group. Auranofin is able to reduce VEGFR3 in a dose-dependent manner in both primary EC and EC cell lines. Interestingly, high concentration of auranofin (≥1 μM) downregulates cellular survival protein thioredoxin reductase (TrxR2), TrxR2-dependent Trx2 and transcription factor NF-κB, whereas it upregulates stress signaling proteins including p38MAPK, leading to EC apoptosis. However, low level of auranofin (≤0.5 μM) specifically induces downregulation of VEGFR3 and VEGFR3-mediated EC proliferation and migration, two critical steps essential for *in vivo* lymphangiogenesis. Mechanistically, auranofin induces VEGFR3 degradation through a lysosome-dependent pathway. Our data show that auranofin-induced downregulation of VEGFR3 is blocked by antioxidant N-acetyl-L-cysteine (NAC) and lysosome inhibitor chloroquine, but is enhanced by proteasomal inhibitor MG132. All above results suggest that auranofin may be a potent therapeutic agent for the treatment of lymphangiogenesis-dependent cancer metastasis [111]. Recently we have also shown that a new anti-cancer agent 1,2-bis(methylsulfonyl)-1-(2-chloroethyl)-2-[(methylamino)carbonyl]hydrazine (laromustine) could inhibit angiogenesis by inhibiting ASK1. Upon decomposition *in situ*, laromustine yields the chloroethylating species 1,2-bis(methylsulfonyl)-1-(2-chloroethyl)hydrazine (90CE) and methyl isocyanate, which could carbamoylate free thiol groups of proteins. 90CE has been shown to kill tumor cells via a proposed mechanism that involves interstrand DNA cross-linking. We show that 1,2-bis(methylsulfonyl)-1-[(methylamino)carbonyl] hydrazine (101MDCE), an analog of laromustine that generates only methyl isocyanate, activates ASK1-JNK/p38 signaling in EC. Mechanistic studies indicate that 101MDCE directly dissociates ASK1 from Trx or indirectly inhibits TrxR to induce EC death through a non-apoptotic (necroptotic) pathway. Our study supports that methyl isocyanates may contribute to the anticancer activity in part by interfering with tumor angiogenesis [112].

## 6. Conclusions and Future Perspectives

Mitochondrial redox homeostasis is an integral component of cells signaling pathways and has been shown to regulate cell transformation, survival, proliferation, invasion, angiogenesis, and metastasis. Like a double-edged sword, the roles of oxidative stress and redox state could exert beneficial or detrimental consequences in various types of cancer cells and tumor microenvironments (Figure 1). Targeting mitochondrial redox-sensitive pathways and transcriptional factors in the appropriate context offers great promise for cancer prevention and therapy, such as mitoTEPO, Auranofin and laromustine. Therefore, further study is required using animal models that critically monitor the roles of oxidative stress and redox state in the possible benefit of antioxidant. As discussed, increased mitochondrial ROS production is critical in cancer cell reprograming by inducing DNA mutations, activation of growth receptor signaling and alterations of mitochondrial genes such as Sirt3. This, however, is often coupled with upregulation of antioxidant system such as NADPH and GSH synthesis, and maintenance of reductive phenotype. This adaptive response to increased ROS production is critical not only for cancerous transformation but also important in maintenance of cancer phenotype. Moreover, this adaptive response may be largely responsible for drug resistance since cancer cells are very well known more resistant to any “oxidant” exposure compared with non-malignant cells, causing inherited problem with standard approach to kill cancer cells by oxidative stress since it leads to tremendous “off-target” non-malignant cells dysfunction and cell death. To improve the potential of translational applications of mitochondria-specific anti-tumor drugs, it is critical to monitor the specific role of oxidative stress and alterations in redox-sensitive signaling pathways in various types of cancer distinct from that is normal cells. It is also pivotal to characterize species, location and kinetics of ROS within cellular and mitochondrial compartments by new small molecule probes and genetically encoded sensors to measure ROS and thiol redox state in living cells and tissues [113,114]. Identification of Sirt3 and other critical regulators that connects mitochondrial ROS, metabolism reprogramming and cancer progression will be of significance in the future. For example, re-expression or re-activation of mitochondrial Sirt3, which is often downregulated in many cancers, may provide potential therapeutic approach. With recent advances in technology of mitochondrial research such as redox proteomics and metabonomics [115,116,117,118], interconnected mitochondrial physiology, metabolism, redox signaling in normal and cancer cells need to be uncovered to properly understand the role of mitochondrial in cancer biology.

## Abbreviations

The following abbreviations are used in this manuscript:

AP-1	activation protein-1
ASK1	apoptosis signal-regulating kinase 1
EC	endothelial cell
ER	Endoplasmic reticulum
Grx2	Glutaredoxin-2
GPx	Glutathione peroxidase
NF-kB	Nuclear factor-kB
OXPHOS	Oxidative phosphorylation
Prx3	Peroxidase-3
ROS	Reactive oxidative species
SOD2	superoxide dismutase
Trx2	Thioredoixn-2
TrxR2	Trx2 reductase-2
